# Electrically induced bacterial membrane-potential dynamics correspond to cellular proliferation capacity

**DOI:** 10.1073/pnas.1901788116

**Published:** 2019-04-18

**Authors:** James P. Stratford, Conor L. A. Edwards, Manjari J. Ghanshyam, Dmitry Malyshev, Marco A. Delise, Yoshikatsu Hayashi, Munehiro Asally

**Affiliations:** ^a^School of Life Sciences, University of Warwick, Coventry, West Midlands, CV4 7AL, United Kingdom;; ^b^Warwick Integrative Synthetic Biology Centre, University of Warwick, Coventry, West Midlands, CV4 7AL,United Kingdom;; ^c^Department of Biomedical Engineering, School of Biological Sciences, University of Reading, Reading, Berkshire, RG6 6AH, United Kingdom;; ^d^Bio-Electrical Engineering Innovation Hub, University of Warwick, Coventry, West Midlands, CV4 7AL, United Kingdom

**Keywords:** bacterial electrophysiology, bioelectricity, cell biophysics, rapid bacterial detection, electrical signaling

## Abstract

Membrane-potential dynamics mediate bacterial electrical signaling at both intra- and intercellular levels. Membrane potential is also central to cellular proliferation. It is unclear whether the cellular response to external electrical stimuli is influenced by the cellular proliferative capacity. A new strategy enabling electrical stimulation of bacteria with simultaneous monitoring of single-cell membrane-potential dynamics would allow bridging this knowledge gap and further extend electrophysiological studies into the field of microbiology. Here we report that an identical electrical stimulus can cause opposite polarization dynamics depending on cellular proliferation capacity. This was demonstrated using two model organisms, namely *Bacillus subtilis* and *Escherichia coli*, and by developing an apparatus enabling exogenous electrical stimulation and single-cell time-lapse microscopy. Using this bespoke apparatus, we show that a 2.5-second electrical stimulation causes hyperpolarization in unperturbed cells. Measurements of intracellular K^+^ and the deletion of the K^+^ channel suggested that the hyperpolarization response is caused by the K^+^ efflux through the channel. When cells are preexposed to 400 ± 8 nm wavelength light, the same electrical stimulation depolarizes cells instead of causing hyperpolarization. A mathematical model extended from the FitzHugh–Nagumo neuron model suggested that the opposite response dynamics are due to the shift in resting membrane potential. As predicted by the model, electrical stimulation only induced depolarization when cells are treated with antibiotics, protonophore, or alcohol. Therefore, electrically induced membrane-potential dynamics offer a reliable approach for rapid detection of proliferative bacteria and determination of their sensitivity to antimicrobial agents at the single-cell level.

## Significance

Transmembrane voltage in bacteria plays a central role both in electrical signaling and cell proliferation. However, whether proliferation state influences the response to electrical signals was unknown. Combining fluorescence time-lapse microscopy with a bespoke device and mathematical modeling, we show that proliferative and inhibited cells respond in opposite directions to an identical electrical signal. The response differentiation can be seen within a minute after stimulation. Therefore, our findings offer an approach for rapid detection of proliferative bacteria at the single-cell level.

Compared with animal bioelectrical signaling, bacterial electrical signaling is understudied and only recently were the excitation dynamics of membrane potential shown to mediate the intra- and intercellular signaling which regulates important physiological processes, namely mechanosensation, spore formation, and biofilm dynamics (1–4). In animal bioelectrical signaling, externally applied electrical stimuli and measurements of cellular electrical properties have been the principle methodology (5–8). This approach has led to many key discoveries regarding the roles of animal bioelectrical signaling [e.g., early tissue development (9, 10), regeneration (11), and carcinogenesis (12–14)] and has fostered the development of real-world applications such as for tissue engineering (15–17), wound healing (6, 18), and electroceuticals (19). Utilizing exogenous stimuli is an important step forward toward understanding bacterial electrical signaling and development of applications based on bacterial electrophysiology. In the past, applications of electric currents to bacteria were used for sanitization (20), electroporation (21), and most recently redox synthetic biology (22). However, due to the only recent discovery of bacterial membrane-potential excitation dynamics, use of external electrical signals in the context of bacterial electrophysiology has been left largely unexplored.

An external electrical stimulus alters cellular membrane potential according to the Schwan equation: ∆*Ψ*_*max*_ = $∆\Psi_{\max}={1.5\mathrm{aE}({1+\left(2\pi f\tau \right)}^{2})}^{-\frac{1}{2}}$, where ∆*Ψ*_*max*_ is the induced membrane potential, *a* is the cell radius, *E* is the applied field strength, *f* is the AC field frequency, and τ is the relaxation time of the membrane (23). This equation, derived from the electromagnetic theory (24), expresses that the maximum change in the membrane potential of a cell caused by an electrical stimulus is proportional to the applied field strength. Theoretically, when an electrical stimulus is applied to proliferative bacterial cells, it should lead to opening of voltage-gated K^+^ channels (Kv) and consequent hyperpolarization due to K^+^ efflux. Substituting the typical values of bacterial resting potential [−140 ∼ −75 mV (25, 26)] and threshold potential for Kv [∼ −50 mV (27)] to the equation, one can expect that the depolarization by an electrical stimulus with the field strength of +35 ∼ 120 mV/µm should open voltage-gated K^+^ channels on bacteria.

In addition to its role in bioelectrical signaling, membrane potential is central to cellular proliferation; it provides the essential driving force for ATP synthesis (28) and is crucial for cell division (29). A quantitative estimation based on the measured energy consumption of *Escherichia coli* suggests that the maintenance of membrane potential accounts for about half of the total energy consumption (30), and thus it is inherently linked to the proliferative capacity, here defined as the capacity to stay out of thermodynamic equilibrium. The proliferative capacity is commonly determined either by direct time-lapse observation of individual cells or by probing the intracellular state using membrane potential. The latter could be achieved using fluorescent indicators for membrane potential such as thioflavin T (ThT), DiOC_2_ (3), and rhodamine 123 (2, 31–34). However, the use of such indicators for determining the proliferative capacity is known to be difficult because membrane potential can be affected by many physiological states and environmental conditions (35–37). Due to the baseline fluorescence being affected by a variety of conditions, comparisons between individual cells, populations of cells, and different species are technically complex. This means that meticulous and tedious calibrations are required for species, strains, media, and detection systems. The difficulties associated with these calibrations often preclude the broad use of these agents for the detection of proliferative bacteria. Nevertheless, these fluorescent indicators provide a useful qualitative measure of intracellular physiological state for live-cell imaging.

The dual roles of membrane potential in both signaling and proliferation prompt the question regarding the interplay between these two roles of membrane potential. In particular, could the electrical response of cells be affected by the proliferative capacity of individual cells? This is an important question for understanding bacterial electrical signaling because the input–output (I/O) relations are fundamental to any “signaling” (38). However, whether cellular responses to an electrical stimulus differ depending on their proliferative capacity remains unclear. If it does, one may expect that an identical signal input produces different outputs depending on their proliferative capacities.

In this study, we utilized an exogenous electrical stimulus to investigate the impact of proliferative capacity on electrical signal response. By experimentally testing a prediction from a mathematical model, we showed that an exogenous electrical stimulus induces hyperpolarization in unperturbed cells while inducing depolarization in inhibited cells. This finding offers an application to use bacterial electrophysiological dynamics for rapid detection of proliferative cells and differentiation of proliferative and nonproliferative cells within a minute after electrical stimulation.

## Results

### Development of an Apparatus That Enables the Monitoring of Membrane-Potential Response to an Exogeneous Electrical Stimulus.

**Fig. 1.**
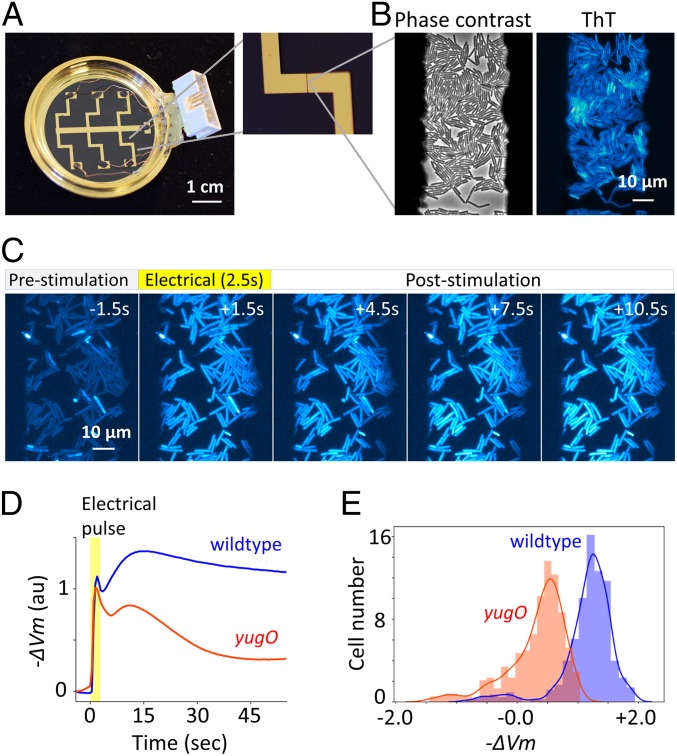
An apparatus enabling concurrent single-cell microscopy and stimulation with exogeneous electrical signal revealed hyperpolarization response to an electrical stimulus. (*A*) Bespoke glass-bottom dish coated with gold-titanium electrodes. Zoomed image on the *Right* shows 50-µm gap between electrodes. Dish is connected to relay circuit to apply electrical stimulation to bacterial cells (see *SI Appendix*, Figs. S1–S3 for details). (*B*) *B. subtilis* cells within the 50-µm electrode gap are visible in phase-contrast and ThT fluorescence images. (*C*) Film-strip images of ThT fluorescence of *B. subtilis* before, during, and after electrical stimulation. Increase in ThT fluorescence indicates hyperpolarization response to an electrical stimulus. (*D*) Mean −ΔVm over time for *B. subtilis* wild-type and *yugO* strains. −ΔVm was calculated by log(*F*_*ThT*_/*F*_*ThT,R*_), where *F*_*ThT*_ is ThT fluorescence and *F*_*ThT,R*_ is ThT fluorescence at resting state (*SI Appendix*). Time traces of individual cells are shown in *SI Appendix*, Fig. S4 (WT, *n* = 321; *yugO*, *n* = 308). Images were taken at 2 fps. (*E*) Histogram of −ΔVm at 30 s after electrical stimulation. The distributions of WT and *yugO* are clearly distinguishable.

To investigate the potential impacts of proliferative capacity on electrical signaling responses, direct observation of cell proliferation, membrane potential, and its response to electrical stimuli at the individual-cell level is needed. However, the commercially available apparatus for neural electrophysiology were unsuitable due to the small size of bacterial cells; i.e., ∼1.4 µm^3^ for *Bacillus subtilis* and *E. coli* (39). To overcome this technical challenge, we designed and developed a tool for bacterial electrophysiology. The tool consists of an electrical relay circuit with an open-source I/O board, Arduino UNO, and a bespoke electrode-coated glass-bottom dish (Fig. 1*A* and *SI Appendix*, Figs. S1–S3, see *Materials and Methods* for details). Bacterial cells were inoculated on agarose pads and placed on the electrode surface. Importantly, this setup enables the monitoring of cells at single-cell resolution using phase contrast and the fluorescence membrane-potential indicator, ThT (Fig. 1*B*).

### Electrical Stimulation Causes Hyperpolarization of Cells via K^+^ Efflux.

To examine whether an externally applied electrical stimulus is capable of opening K^+^ channels on bacterial membranes, we applied an exogenous electrical stimulus (60 mVpp/µm AC 0.1 kHz for 2.5 s) to *B. subtilis* cells placed between the 50-µm electrode gap (Fig. 1*B*). Upon electrical stimulation, the intensity of ThT fluorescence increased, indicating a hyperpolarization response (Fig. 1*C*). Single-cell analysis of the fluorescence dynamics revealed that most cells exhibited the hyperpolarization of membrane potential (V_m_), while a small subpopulation of cells depolarizes upon stimulation (*SI Appendix*, Fig. S4*A*). Intriguingly, the cell elongation rate of these depolarizing cells was found to be much lower compared with other cells (*SI Appendix*, Fig. S5). No significant change in ThT intensity was observed with the absence of electrical stimulus (*SI Appendix*, Fig. S6) or mild change in pH (*SI Appendix*, Fig. S7), indicating that the observed dynamics are induced by the electrical stimulus. The hyperpolarization response suggests that electrical stimulation causes the efflux of cations such as K^+^, the dominant intracellular cation. It has been shown that chemical depolarization opens the YugO potassium channels (2). The Schwan equation predicts that external electrical stimuli can depolarize bacterial cellular membranes (40). Therefore, we hypothesized that depolarization by the electrical stimulation opens YugO channels, resulting in membrane hyperpolarization by K^+^ efflux.

To test this hypothesis, the same experiment was conducted with a mutant strain lacking the gene encoding the YugO potassium channel. Although the strain still showed an initial hyperpolarization response in the timescale of ∼5 s, the hyperpolarization response on the timescale of ∼30 s is greatly attenuated (Fig. 1 *D* and *E* and *SI Appendix*, Fig. S4*B*). For a further test, we measured the intracellular K^+^ levels using Asante Potassium Green-2 AM (APG-2 AM) (2), and found that the intracellular K^+^ decreases upon stimulation (*SI Appendix*, Fig. S8). These results suggest that K^+^ efflux through the YugO channel is responsible for the hyperpolarization following an electrical stimulation. It also suggests that there may be other voltage-gated channels with faster timescales of activation and inactivation (∼5 s). It is worth noting that bacteria have several voltage-gated ion channels (4, 41, 42). This is an interesting observation in conjunction with the fact that different neural ion channels have their unique timescales of activation and inactivation which contribute to information processing (27). We also conducted the same experiment with *E. coli* and confirmed that *E. coli* cells also exhibit hyperpolarization in response to an external electrical stimulus (*SI Appendix*, Fig. S9). Together, these results demonstrate with single-cell resolution that a pulsed electrical stimulus can induce a hyperpolarization response in bacterial cells.

### Exposure to UV-Violet Light Abolishes Hyperpolarization Response to an Electrical Stimulus.

**Fig. 2.**
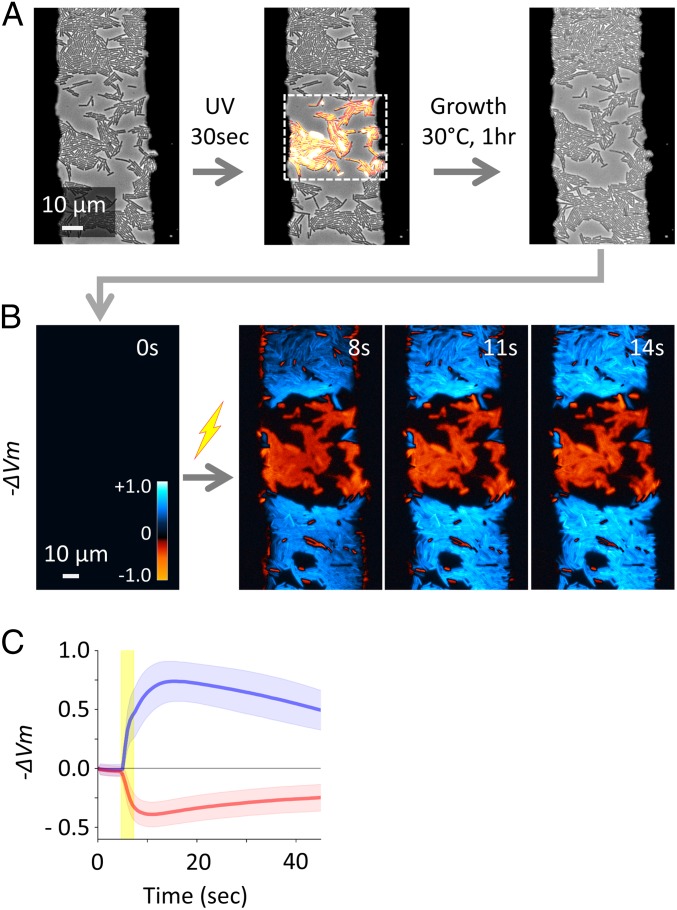
UV-V irradiation makes *B. subtilis* cells respond to an electrical stimulus in the opposite direction. (*A*) Phase-contrast microscopy image shows WT *B. subtilis* cells within the electrode gap. A rectangular region indicated by the dashed line within the field of view was irradiated by UV-V light. Growth was suppressed in the UV-V–irradiated region, while cells outside of the UV-irradiation region replicated. (*B*) The region shown in *A* was treated with an electrical stimulus. −ΔVm was calculated from ThT fluorescence [log(*F*_*ThT*_/*F*_*ThT,R*_)] and shown with the colormap in the panel. To an identical electrical stimulus, unperturbed cells hyperpolarized (blue) and UV-V–irradiated cells depolarized (red). (*C*) Mean (thick lines) and SD (shaded color) of −ΔVm for cells in unperturbed (blue) and UV-V–irradiated (red) regions.

Having tested our apparatus, we examined the impact of proliferative capacity on signal response by using inhibited cells. To inhibit the proliferative capacity of cells, we chose UV-Violet light (400 nm) because it is one of the most commonly used sanitization methods, which has been shown to be effective with both Gram-positive and Gram-negative bacteria (43, 44). Importantly, application of UV-V light allows spatially precise inhibition, creating both irradiated and unirradiated regions within the same field of view. This is critical because it ensures that an identical electrical stimulation is applied to both proliferative and inhibited cells. We irradiated *B. subtilis* cells in a defined region by UV-V light for 30 s (Fig. 2*A*). The growth suppression of the irradiated cells was confirmed by the single-cell analysis of phase-contrast time-lapse microscopy before being stimulated with an electrical pulse (*SI Appendix*, Fig. S10). Upon an electrical stimulation, the irradiated cells exhibit depolarization, while cells in untreated regions become hyperpolarized, despite the fact that both received an identical electrical stimulus (Fig. 2 *B* and *C*). This experiment demonstrates that an electrical stimulus can result in cellular response in apparent opposite directions depending on whether cells are exposed to UV-V or not. Strikingly, analysis of the fluorescence dynamics after electrical stimulation showed a clear bimodal distribution correlating with the irradiation (*SI Appendix*, Fig. S11 *A* and *B*). To examine whether this is unique to *B. subtilis*, we conducted the same experiment with *E. coli* cells. The result with *E. coli* also revealed distinct responses depending on whether cells were treated by UV-V or not (*SI Appendix*, Fig. S11*C*). These results suggest that proliferative and growth-inhibited cells respond differently to an identical electrical stimulus and that this difference in response dynamics is common to these two phylogenetically distant model organisms.

### A Mathematical Model Suggests That the Response Differentiation Is Due to the Shift in Resting Membrane Potential.

**Fig. 3.**
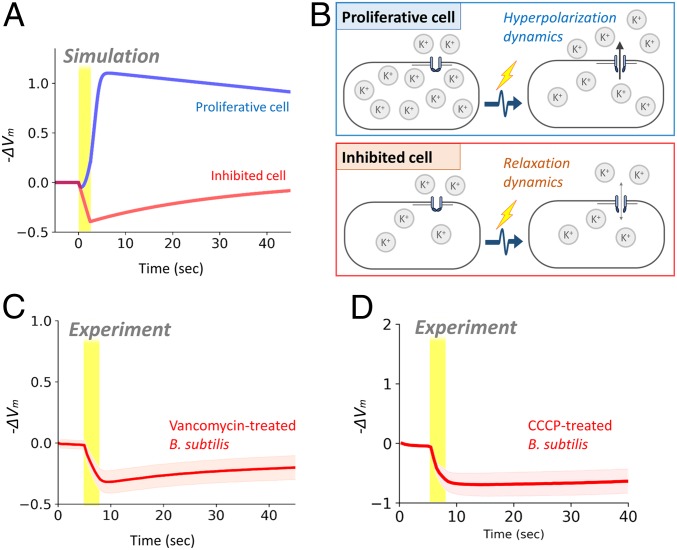
Shift in resting-state membrane potential is sufficient to describe the distinct responses between proliferative and inhibited cells. (*A*) Numerical simulation of the FHN bacteria model, corresponding to Fig. 2*C*. Despite being stimulated by an identical electrical stimulus, proliferative cells (blue) hyperpolarize and inhibited cells (red) depolarize. See *SI Appendix* for model details. (*B*) Illustration of the biological mechanism of the response differentiation between proliferative and inhibited cells. (*C* and *D*) Time trace of membrane potential change (−ΔVm) with *B. subtilis* cells exposed to (*C*) vancomycin or (*D*) CCCP. Shading shows SD.

To gain conceptual understanding of the observed distinct responses to an identical electrical stimulus, we used a mathematical framework based on the FitzHugh-Nagumo (FHN) neuron model. The FHN neuron model, originally published over half a century ago (45), is one of the most paradigmatic models in neuroscience due to its mathematical simplicity and richness for capturing complex behaviors (46). We extended the FHN neuron model to bacterial electrophysiology while retaining its mathematical simplicity (*SI Appendix* for details). Briefly, in our FHN bacteria model, we considered two parameters representing the resting-state membrane potential and K^+^ transmembrane gradient. Numerical simulations of the model showed that an external electrical stimulus causes hyperpolarization in proliferative cells, while the same stimulus produces a relaxation response from depolarization in inhibited cells (Fig. 3*A* and *SI Appendix*, Fig. S12). This is because the direction of K^+^ flux (influx or efflux) differs depending on the resting-state membrane potential and transmembrane concentration gradient of K^+^ (Fig. 3*B*). According to our simulations, opening of K^+^ channels in proliferative cells results in K^+^ efflux following the concentration gradient, thus causing hyperpolarization. However, the same opening of K^+^ channels only leads to the relaxation from depolarization due to weaker transmembrane K^+^ gradient. This mechanistic insight from the simulations predicts that the shift in resting-state membrane potential is sufficient to alter the response dynamics to an electrical stimulus. This means that different classes of growth-inhibition treatments should also make cells respond by depolarization.

To examine this prediction from the model, we conducted the electrical stimulation experiment with the cells exposed to different classes of common growth-inhibition treatments; namely, an antibiotic vancomycin, a protonophore carbonyl cyanide m-chlorophenyl hydrazone (CCCP), and a common antimicrobial agent ethanol. As predicted by the model, vancomycin-treated cells exhibited a clear depolarization in response to an exogenous electrical stimulus (Fig. 3*C*), as opposed to the hyperpolarization response seen in unperturbed cells (Fig. 1*C*). Experimental tests with the cells treated with ethanol or CCCP also showed depolarization response (Fig. 3*D* and *SI Appendix*, Fig. S13). These results strongly support the conclusion drawn from the FHN model that general stress treatments alter the cellular response to an electrical stimulus. Conversely, this finding suggests that the proliferative and inhibited cells can be distinguished based on their response to an exogeneous electrical stimulus.

### Selective Antibiotics Enable Classification of Coliforms in a Mixed Culture.

The above observations and understanding raised the possibility of using the electrically induced membrane-potential dynamics for rapid detection of proliferative cells in a mixed-species culture. Such application would be significant to industries and medical sectors as it can offer rapid single-cell level detection of proliferative cells of biological samples. For proof of concept, we explored this possibility using a coculture of *E. coli* (gram-negative) and *B. subtilis* (gram-positive). Our hypothesis was that the above approach of measuring electrically induced membrane-potential dynamics combined with exposure to vancomycin allows for the rapid differentiation of *E. coli* and *B. subtilis*. This is based on the fact that vancomycin inhibits the cell-wall synthesis of gram-positives but is largely inactive to gram-negatives due to their outer membrane barrier (47).

**Fig. 4.**
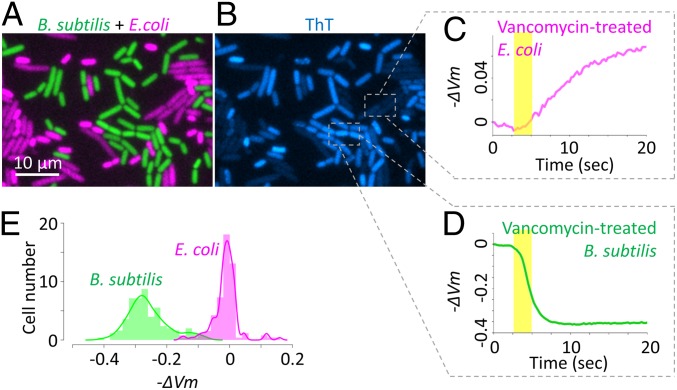
When treated with vancomycin, *B. subtilis* and *E. coli* cells can be differentiated based on their response to an electrical stimulus. (*A*) Microscopy image showing the coculture of fluorescently labeled *E. coli* (magenta) and *B. subtilis* (green) cells treated with vancomycin. (*B*) ThT fluorescence image at the corresponding region. (*C* and *D*) Time trace of −ΔVm, calculated from ThT fluorescence intensity, of the regions defined in *B*. (*E*) Histogram of −ΔVm at 10 s after electrical stimulation of *B. subtilis* and *E. coli* shows clear differentiation of peaks with the presence of vancomycin.

To test this hypothesis, we cocultured fluorescently labeled strains of *E. coli* and *B. subtilis* expressing mCherry and YFP under IPTG-inducible promoters for *E. coli* and *B. subtilis*, respectively. Multichannel fluorescence imaging exhibited distinct signals for *E. coli* and *B. subtilis* (Fig. 4*A*). The fluorescence of ThT showed lower intensity with *E. coli* compared with *B. subtilis* (Fig. 4*B*). Although the exact reason for this is unclear it may suggest that there is a difference in stability or fluorescence yield of ThT between these two species. This difference in intensity highlights an advantage of our approach focusing on the response dynamics rather than being reliant on the initial affinity of the cells for the dye. After an hour-long exposure to vancomycin, the coculture was treated with an external electrical stimulus. The result revealed distinct patterns of membrane-potential dynamics for *E. coli* and *B. subtilis* cells treated with vancomycin. Specifically, the electrical stimulation causes *E. coli* to become hyperpolarized, while depolarizing vancomycin-treated *B. subtilis* (Fig. 4 *C* and *D* and *SI Appendix*, Fig. S14). Similar patterns were observed with monocultures of *E. coli* or *B. subtilis* (*SI Appendix*, Fig. S15). We also confirmed that vancomycin is active on *B. subtilis*, but not on *E. coli* (*SI Appendix*, Fig. S16). The histogram of individual-cell response revealed distinct distributions between *E. coli* and *B. subtilis* (Fig. 4*E*). These results thus demonstrate that our approach can be combined with selective culture methods that are commonly used for species differentiation.

## Discussion

In this study, we developed and demonstrated an experimental tool for studying the input–output relation of bacterial electrical signaling. Using this tool combined with time-lapse microscopy, we showed that proliferative and inhibited cells respond to an electrical stimulation in apparent opposite directions. A mathematical model suggested that the apparent opposite responses can arise from diverse antibacterial stresses. We experimentally confirmed this using UV-V light, antibiotics, ethanol, and CCCP. The distinct response dynamics between proliferative and inhibited cells offer a rapid single-cell detection of proliferative cells, which can be combined with differentiation techniques using selective growth media.

Bacterial electrical signaling mediated by membrane-potential dynamics regulates various physiological processes, such as metabolic coordination in biofilms and mechanosensation (4, 48). However, it remains unclear how cells decode various information from the received electrical signal. We demonstrated that proliferation capacity adds to the complexity of bacterial electrical signaling, which presents a clue for elucidating the signal-decoding mechanism. Recent study showed that only a subpopulation of cells contributes to the biofilm electrical signaling, which optimizes the cost–benefit tradeoff of signal transmission (49). Cell-to-cell heterogeneity in electrical signaling is also observed in mechanosensation (4), sporulation (3), and antibiotics stress response (50). These heterogeneities are important for understanding how cells decode electrical signal inputs. However, the mechanisms by which these heterogeneities emerge remain unclear. Our finding raises a possibility that the variations in proliferative capacity may underpin the signaling heterogeneities since the bidirectional interaction between metabolisms and electrical signaling could in theory induce multistability in the system. We expect that future mathematical and computational studies, combined with single-cell analyses, will investigate the input–output relation and information processing of bacterial electrical signaling.

We showed, through the combination of simulations and experiments, that the distinct response dynamics between proliferative and inhibited cells can be described by the shift in resting membrane potential. Since the maintenance of membrane potential accounts for a major fraction of cellular energy consumption (30), it seems plausible that different types of metabolic and environmental stress would all ultimately lead to imbalance in transmembrane ion gradient at resting state. For instance, the Ktr potassium uptake system in *B. subtilis* and the Kdp potassium uptake system in *E. coli* are both ATP driven (51, 52). This means that maintaining the resting-state membrane potential requires constant consumption of ATP to keep the intracellular K^+^ level up to two orders of magnitude higher than the outside. When an electrical stimulus opens voltage-gated K^+^ channels, the flux of K^+^ through the channel follows the electrochemical gradient of K^+^, which indicates that hyperpolarization due to K^+^ efflux occurs only when intracellular K^+^ concentration is significantly greater than the extracellular level. This effect was accounted for by the parameter *k*_*K*_ in our FHN bacteria model (*SI Appendix*). Using the tool, we will be able to carry out future studies to quantitatively analyze the dynamics of other electrophysiologically important ions, such as Ca^2+^ and Cl^−^ and determine their contributions to the membrane-potential dynamics in bacteria. Biological and biophysical characterization of the dynamics of transmembrane gradients of different ions and their corresponding channels will form the fundamental basis to our understanding of bacterial electrical signaling. Another important area of research is to identify the molecular mechanism by which electrical stimuli open the YugO potassium channel.

We hope that the experimental setup developed and demonstrated in this work will encourage more microbiologists to consider bacterial electrophysiology for gaining new insights into their physiological processes of interest. Although it is known that membrane potential is closely associated with important microbiological processes, including persister formation and antibiotics resistance (53–56), biological and mechanistic insights into such relation are largely limited. This is partly because of the shortage of appropriate experimental tools for molecular microbiology investigations. Recent discoveries of various signaling roles for bacterial membrane potential and ion flux (1, 2, 50, 57) suggest that bacterial electrical signaling may play roles in many more physiological processes than previously realized. The uses of exogeneous electrical stimuli should unlock opportunities to gain new biological insights regarding signaling roles of membrane-potential dynamics. In parallel, it will also facilitate the development into new synthetic-biology technologies for electrical control and bioelectrical engineering of bacterial functions.

Finally, our findings offer an approach for rapid detection of proliferative bacteria without the need for observing actual proliferation or the time-consuming calibrations for bacterial species. The growing demand for fast identification of live bacterial cells has been driving the development of novel technologies for rapid bacterial detection (58). Our approach could detect proliferative cells within a minute after an electrical stimulation, as opposed to the typical duration of 12–48 h required by conventional culture-based detection methods (58). This attractive feature could accelerate the examination of antimicrobial agents and diagnosis in the medical sector and enable efficient quality control in the water, pharmaceutical, food, and beverage industries. The capability to differentiate UV-damaged cells from healthy cells is also unique. In further studies, we will examine if this approach enables the detection of medically and industrially relevant bacterial species for timely diagnosis. If applied widely, this approach of using membrane-potential dynamics and exogenous electrical stimuli could bring great societal benefits by accelerating the detection of proliferative bacteria and determination of their sensitivity to antimicrobial agents.

## Materials and Methods

### Strains and Growth Conditions.

*E. coli* and *B. subtilis* cells were routinely grown in lysogeny broth (LB) or on an LB agar [1.5% (wt/vol)] plate. The reporter and mutant strains used in this study are listed in *SI Appendix*, Table S1. For electrical stimulation experiments, a colony from an LB agar plate was inoculated into liquid LB and subsequently incubated at 30 °C with aeration (200 rpm; model 311DS, Labnet) to OD_600_ ∼ 1.5. Cells were then resuspended in minimal salts glutamate glycerol (MSgg) media (59): 5 mM potassium phosphate (pH 7.0), 10 mM Mops (pH 7.0), 2 mM MgCl2, 700 µM CaCl2, 50 µM MnCl2, 100 µM FeCl3, 1 µM ZnCl2, 2 µM thiamine-HCl, 0.5% (vol/vol) glycerol, and 0.5% (wt/vol) monosodium glutamate. Note that the Mops concentration is reduced by 10-fold from the original receipt of MSgg to suppress electrolysis of the media. After a 1-h incubation in liquid MSgg, cells were inoculated onto MSgg low-melting point (LMP) agarose pads containing 10 µM ThT (Sigma-Aldrich). With experiments focusing on mixed culture, 5 µg/mL vancomycin hydrochloride, 1 mM NH4Cl, and 0.25% (wt/vol) glucose were supplemented to the MSgg LMP agarose pads. Pads were prepared as described previously (60). Briefly, LMP agarose (Formedium, bacteriological granulated agar) was dissolved in MSgg and left to solidify between two 22 mm × 22 mm cover glasses (Fisher Scientific) for 10 min at room temperature. When stated, final concentration of 1% (vol/vol) ethanol or 100 µM CCCP was supplemented to MSgg liquid and MSgg agarose pads. For the measurements of intracellular K^+^, APG-2 (Abcam PLC), instead of ThT, was supplemented to MSgg agarose pads at the final concentration of 2 µM. The solidified agar was cut into ∼5 mm × 5 mm pads. A total of 2 µL of bacterial liquid LB culture (OD_600_ ∼ 1.5) was inoculated onto each pad. Pads were then placed on the gold-coated glass-bottom dish for microscopy.

For the construction of *E. coli* K12 pGEX6P1-mCherry strain, pGEX-6P1 plasmid (GE Healthcare) was digested with EcoRI and NotI restriction enzymes (New England Biolabs). The mCherry gene was amplified by PCR using PrimeSTAR Max DNA Polymerase (Takara Bio) using the primers AP609 (5′-CCCCTGGGATCCCCGGAATTCATGGTGAGCAAGGGCGAG-3′) and AP610 (5′- AGTCACGATGCGGCCGCTCGAGTTTAGCACTTGTACAGTTCGTCCATG-3′). The PCR product was assembled together with the digested pGEX-6p1 by the Gibson Assembly using Gibson Assembly Master Mix kit (New England Biolabs) and transformed into competent *E. coli* K12 cells. The competent cells were prepared using Mix&Go Competent Cells kit (Zymo Research). The sequence of the assembled plasmids was confirmed by Sanger sequencing (Source BioScience) and aligned using Benchling (https://benchling.com/).

### Electrical Stimulation.

Application of electrical stimulation was accompanied with time-lapse imaging with 2 frames per second (fps) for 1 min. An alternating current (AC) signal [0.1 kHz; 3 V peak-to-peak (−1.5 ∼ +1.5 V)] was generated using an arbitrary function generator (Tektronics) and connected to a series of relays, each corresponding to an electrode on the gold-coated dish (*SI Appendix*, Fig. S2). The camera trigger was connected to Arduino UNO R3 in the relay circuit to control the timing of electrical stimulation; upon counting 10 camera exposures, the relay to the electrode being imaged opened for 2.5 s, applying electrical stimulation to the electrode while simultaneously imaging.

### Time-Lapse Microscopy.

The membrane-potential dynamics and growth of individual cells were recorded using an inverted epifluorescence microscope, DMi8 (Leica Microsystems), operated by MetaMorph (Molecular Devices). The microscope is equipped with an incubation chamber (i8 Incubator; Pecon) which maintained the temperature at 30 °C throughout the experiments. Before microscopy experiments, the chamber was set to 30 °C for at least 3 h, and samples were placed in the chamber for 1 h. For all observations, a 100× objective lens (N.A. = 1.3, HCX PL FLUOTAR; Leica) was used and images were taken with scientific cMOS camera ORCA-Flash 4.0 v2 (Hamamatsu Photonics). Cell growth was monitored using phase contrast (exposure time: 100 ms). ThT fluorescence was detected using a single-band filter set consisting of excitation filter (Ex) 438/24 nm, emission filter (Em) 483/32 nm, and dichroic mirror 458 nm (Semrock), with exposure time of 150 ms. For the mixed culture experiment, YFP was detected using a filter set consisting of Ex 509/22, Em 544/24, and dichroic mirror 526 (Semrock). mCherry was detected using a filter set consisting of Ex 554/23, Em 609/54, and dichroic mirror 573 (Semrock). The exposure time for the imaging of both YFP and mCherry was 300 ms.

For irradiation to UV violet light, cells were irradiated by UV-Violet light for 30 s using the inverted microscope DMi8 (Leica Microsystems) and the LED light source, SOLA SM II Light Engine (Lumencor), with an excitation filter 400/16. Field diaphragm of the microscope was used to irradiate only a specific region of the field of view. Before electrical stimulation, a 1-h growth period was allowed during which cells were observed using phase-contrast microscopy to ascertain the effects of UV-V light. For ethanol experiments, MSgg containing 1% (vol/vol) ethanol was used instead of Msgg.

## Supplementary Material

Supplementary File
