# Supplementary material for: Electrically induced bacterial membrane-potential dynamics correspond to cellular proliferation capacity
Source: Proc Natl Acad Sci U S A. 2019 Apr 18;116(19):9552–7. doi: 10.1073/pnas.1901788116 (PMC6511025; doi:10.1073/pnas.1901788116)
Supplement: Supplementary File [file pnas.1901788116.sapp.pdf]

PNAS
www.pnas.org

Supplementary Information for

Electrically induced bacterial membrane potential dynamics correspond to cellular proliferation capacity

James P Stratford, Conor L A Edwards, Manjari J Ghanshyam, Dmitry Malyshev, Marco A Delise, Yoshikatsu Hayashi and Munehiro Asally

Munehiro Asally
Email: m.asally@warwick.ac.uk

This PDF file includes:

SI text
Figs. S1 to S16
Table S1 to S2
References for SI reference citations

www.pnas.org/cgi/doi/10.1073/pnas.1901788116

Supplementary Information Text

FHN bacteria Model

Our model is extended from the FitzHugh-Nagumo (FHN) neuron model. As mentioned in the main text, the FHN neuron model is extensively studied due to its capability of capturing non-linear behaviors including the excitation and relaxation dynamics of firing neurons. The FHN neuron model consists of only two variables; namely, membrane potential (V_m) and recovery variable (W). When an external stimulus above a threshold is applied to the FHN neuron system, it can exhibit a characteristic excursion in the phase space of V_m and W. This excursion in the phase space recapitulates the excitation of neurons, e.g. all or none depolarization of the membrane potential for a given strength of stimulation.

To recapitulate the experimental results of bacterial membrane-potential dynamics, we developed the FHN bacteria model as follows;

$$\frac{dV_m}{dt} = k_K((V_m + V_{m,0}) - \alpha(V_m + V_{m,0})^3 + W) + \frac{dI_v}{dt}$$

$$\frac{dW}{dt} = -((V_m + V_{m,0}) + \beta - W) + \frac{dI_w}{dt}$$

where k_K represents the degree of transmembrane electrochemical gradient of potassium ions, $V_{m,0}$ is the basal shift in membrane potential, α is a parameter that control the dynamic range of membrane potential and I is exogeneous electrical stimulus. We note that the FHN bacteria model and the FHN neuron model are mirror symmetric to each other when plotted in the phase space of V_m and W. This accounts for the hyperpolarization, rather than depolarization, describing the excitation dynamics of bacterial membrane potential dynamics. β is a parameter that determines the resting-state membrane potential ($V_{m,R}$). Considering that potassium ions are the major contributor to the resting-state membrane potential, β is defined as the follows;

$$\beta = 1 - 0.1 \log k_K$$

Proliferative and inhibited cells were accounted by k_K. Mathematically, k_K adjusts the evolution timescale of V_m; small k_K gives rise to slow dynamics of V_m. All other parameters are set to the same for proliferative and inhibited cells. To simulate the exogeneous electrical stimulation experiments, an electrical stimulation (I_v, I_w) was transiently applied to the equilibrium state of the FHN bacteria system. The dynamics of V_m after a transient stimulus were numerically simulated with the above coupled ordinary differential equations.

We used the Nernst equation to allow comparison of the simulation results and experimental data of ThT fluorescence intensity (F_i). According to the Nernst equation, the membrane potential in the unit of mV (millivolt) at 37°C is written as;

$$V_m = -58 \log_{10} \frac{F_i}{F_o}$$

Defining $V_{m,R}$ as the resting-state membrane potential. We consider the change in membrane potential ($-\Delta V_m$) as;

$$-\Delta V_m = V_m - V_{m,R}$$

Putting Nernst equation into this, we get;

$$-\Delta V_m \propto \log_{10} \frac{F_i}{F_{i,R}}$$

where $F_{i,R}$ is the intracellular ThT fluorescence level at resting state. Therefore, the calculation of ΔV_m allows the comparison of experimental and simulation results.

All parameters used for numerical simulations are listed in Table S2. The simulation results of the ΔV_m over time are shown in Fig. 3. With the parameters for the proliferative cells, the system exhibit hyperpolarization even though the external stimulus was applied to the direction of depolarization. In order to understand this excitation dynamics, the dynamics in the phase space of V_m and W is shown in Fig. S8. This figure shows that the excitation response of the cells indeed corresponds to a large excursion after the stimulation on the depolarized direction. When k_K is shifted, the response demonstrates a relaxation process. These simulation results recapitulated the experimental results showing the apparent opposite responses to an identical stimulation (Fig. 2). These dynamics are qualitatively different behaviors, namely excitation and relaxation. When k_K is very small, time derivative of V_m becomes non-excitable since the velocity field in Vm-W plane becomes dominated by the dynamics of W.

Table S2

Simulation parameters	
$V_{m,0}$	1.5
k_K (inhibited cell)	0.1
k_K (proliferative cell)	10
α	10
I_v	0.01
I_w	-0.075

Nernstian chemicals

Nernstian chemicals are widely used as indicators for membrane potential (1). ThT has been used as a Nernstian dye which report the membrane potential (2–4). This means the transmembrane distribution of ThT at equilibrium follows the Nernst equation:

$$V_m = \frac{RT}{zF} \ln \frac{c_e}{c_i}$$

where V_m is membrane potential in millivolt, R is the universal gas constant, T is the absolute temperature, z is the ionic charge, F is the Faraday constant, c_e and c_i are the external and internal ion concentrations respectively. Rearranging this equation, the intracellular concentration of ThT (c_i) can be determined as a function of membrane potential.

$$C_i = \frac{C_o}{e^{\frac{zFV_m}{RT}}}$$

In our experiments, we used 10 µM ThT in the medium. Substituting the known values into the equation, we reach the intracellular concentration of ThT (c_i) in µM as:

$$C_i \approx 10^{(\frac{V_m}{61.5}-1)}$$

Therefore, the intracellular concentration of ThT can be solely determined by a function of membrane potential (V_m). Therefore, while permeability of ThT could alter the time it reaches to the equilibrium, it is expected not to have an impact on the equilibrium intracellular concentration of ThT.

Materials and Methods

Relay circuit

Components shown in Fig. S3 were mounted through-hole onto a solderable breadboard with copper tracks printed onto one side. Connections between components were accomplished by soldering jumper wires of appropriate length to the appropriate position on the copper track. A track breaking tool was used to isolate the components which should not be connected but were on the same track. The components were assembled as shown in the circuit diagram (Fig. S3). The trigger input from scientific cMOS camera (Orca Flash 4.0, Hamamatsu) was connected to the circuit through a BNC (Bayonet Neill-Concelman) connector soldered onto the circuit board. The signal pin from the BNC connector was further connected to an analogue pin on an Arduino UNO R3 (Arduino, arduino.cc) to record when the voltage output from the camera attached to the microscope. A counting loop was implemented in Arduino script to count camera exposures to coordinate the electrical stimulate and sequential imaging. At the programmed time in the imaging sequence a digital pin is set 'HIGH' to provide power to an electro-mechanical relay. This switches the relay throw from ground to the input signal from the arbitrary function generator (Tekronix). All counter electrodes are permanently grounded through a common ground connection.

Constructing Bespoke Electrode Dishes

Stainless steel disks of 50 µm in thickness and 39 mm in diameter were manufactured by Laser Micromachining Ltd. with a negative electrode design and used as coating masks. A negative mask was placed into a glass-bottom dish (HBST-5040, Willco Wells). The glass-bottom dish with a mask in place was then attached to 40 mm stainless steel washers on a silicon wafer using several 5 mm neodymium magnets. Once attached, the silicon wafer was mounted in an Electron Beam Physical Vapor Deposition System (A custom built model II2000EB, Scientific Vacuum Systems); a 20 nm titanium adhesion layer and a 100 nm gold conductive layer were deposed onto the dish, with the mask permitting the electrode design to be applied. Copper wire was soldered to corresponding connections on a 10 pin IDC (Insulation-displacement connector, RS Components) at channels corresponding to the electrodes. Small holes were made through the plastic rim of the dish, with the copper wire being threaded through until all were in their corresponding positions. The IDC was then fixed in position using two-part waterproof adhesive (Araldite Rapid, RS Components). Once the IDC was fixed in place the wires were attached to their corresponding electrode terminals with aqueous graphite conductive adhesive (Alfa Aesar), then left overnight at room temperature to set to reduce resistance of the adhesive. Following this, the graphite connections were covered with Araldite adhesive to protect them from moisture degradation.

Image analysis

Region of interest (ROI) was registered for individual cells and the fluorescence intensity over time for each ROI was measured using Fiji/imageJ. The results were saved in csv (comma-separated values) and imported to python. Based on the Nernst equation (*See SI text* for details), $-\Delta V_m$ was calculated by taking the logarithms of the ratio between intracellular ThT fluorescence intensity (F_i) and the intensity at the resting state ($F_{i,R}$). Cell elongation rate was measured as an indicator of proliferation capacity since it is associated with cell growth rate(5, 6). For measurement of cell elongation rate, ROI was registered for individual cells and the cell size over time for each ROI was measured. The results were saved in csv and imported to MATLAB. The elongation rates were determined as the slope of fitted straight lines. Numerical calculations were performed in python using scipy (scipy.org) and numpy (numpy.org). Graphs and plots were generated using the python data-visualization packages matplotlib (matplotlib.org) and seaborn (seaborn.pydata. org). Scripts were written using Jupyter Notebook (jupyter.org/).

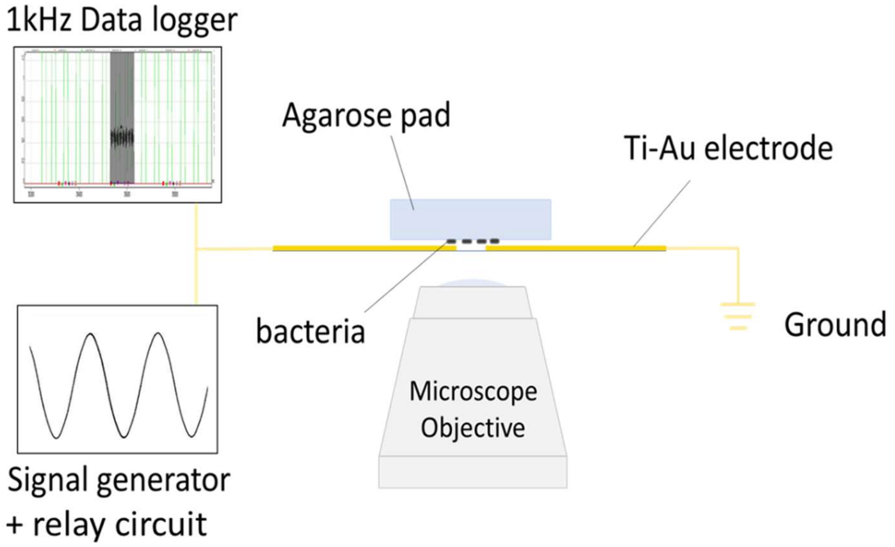

Figure S1 Schematic diagram of the experimental setup. The Ti-Au electrode coated dish (Fig. 1A) is connected to a data logger, signal generator and a relay circuit. Bacteria are inoculated on an agarose pad and placed on electrode-coated dish.

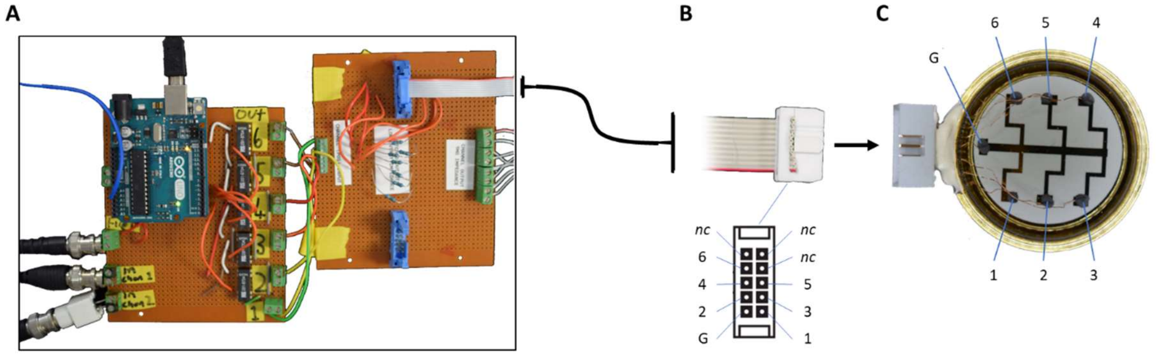

Figure S2 Schematic images of relay circuit and electrode-coated dish. A) Photograph of the custom-built relay circuit with Arduino UNO R3. B) The pin layout of 10-pin IDC output was used to connect the relay circuit and dish. Numbers correspond to each position shown in panel C. nc is not-connected. C) The electrode-coated dish image with the labels of corresponding terminals of each pin. This provides an individual control of firing electrodes.

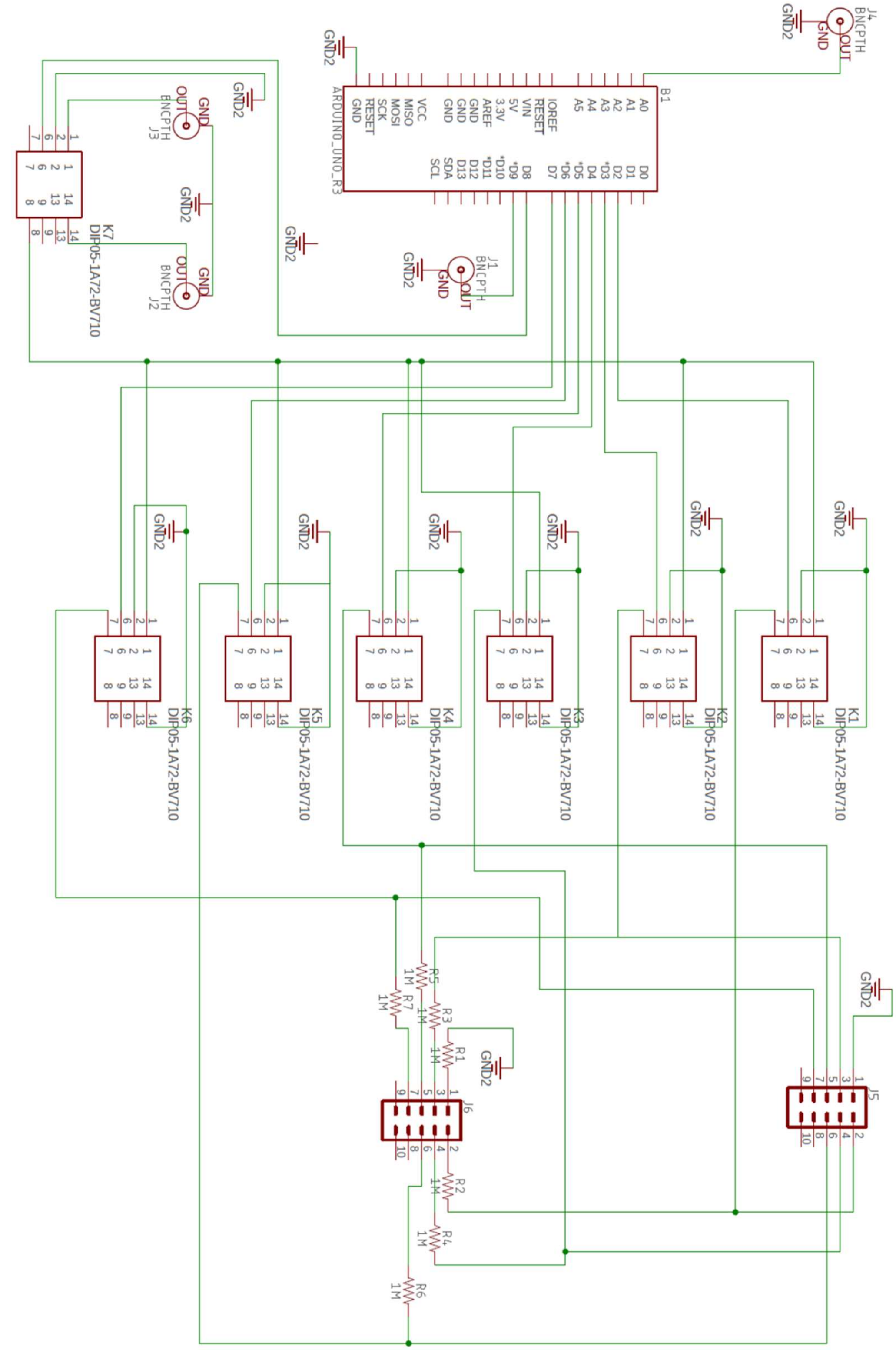

Figure S3 Schematic diagram of relay circuit. Schematic diagram of the relay circuit with Arduino UNO. K1-6 correspond to channels shown in Fig. S2.

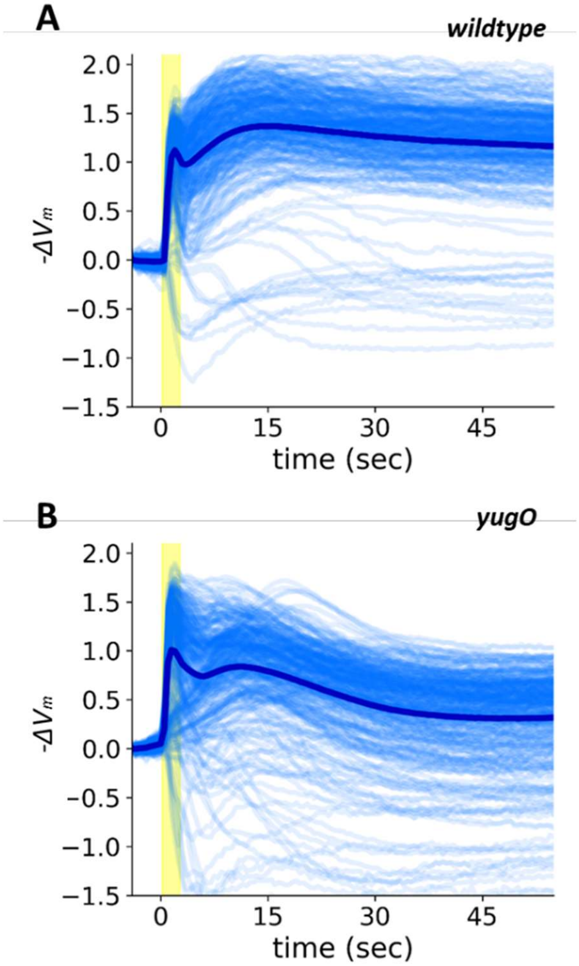

Figure S4 Single-cell time traces of membrane-potential response to an electrical stimulus. Membrane potential dynamics of A) wildtype and B) *yugO B. subtilis* strains. -ΔVm = log(I_t/I_0), where *I* is ThT intensity.

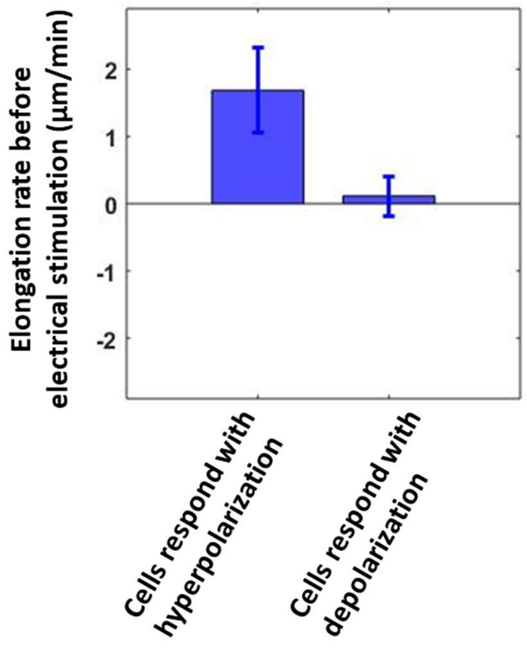

Figure S5 Growth of the cells respond with depolarization is halted before electrical stimulation. Mean elongation rates before electrical stimulation were quantified for the individual *B. subtilis* cells. (n = 15 cells for each). Error bars are standard deviation.

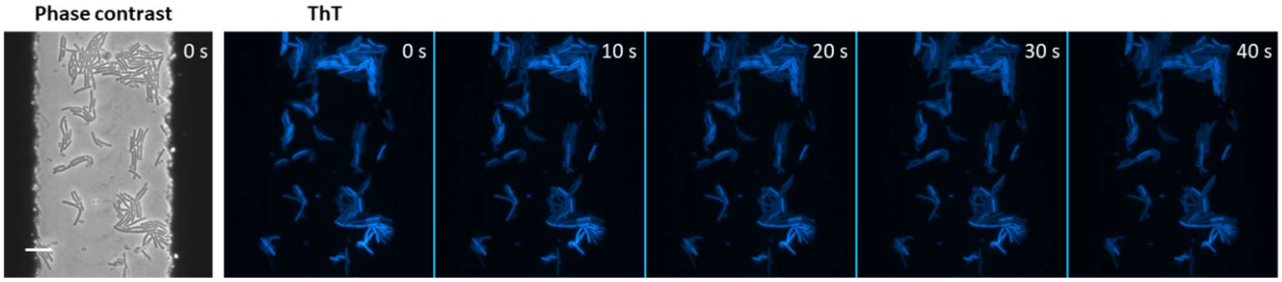

Figure S6 Resting-state membrane potential is static. Film-strip images of ThT fluorescence of *B. subtilis* cells without electrical stimulation. No significant changes were observed without electrical stimulation.

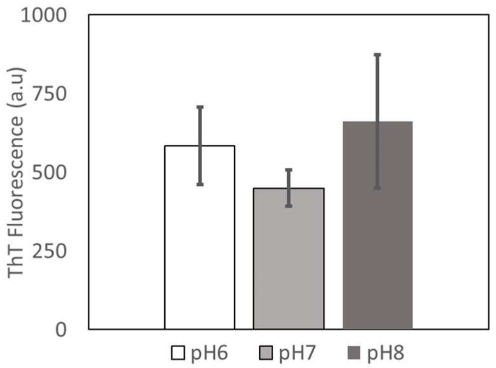

Figure S7 Mean ThT fluorescence intensity of *B. subtilis* cells at different pH are similar. ThT intensity was measured at single-cell level at different pH (n = 60 cells for each). Error bars are standard deviation.

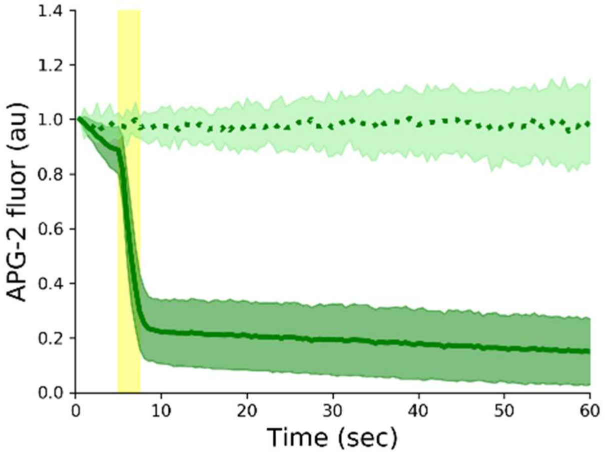

Figure S8 Electrical stimulation causes K+ efflux. Mean intracellular potassium levels measured with APG-2 AM with and without electrical stimulation with *B. subtilis*. Solid green line is the mean APG-2 intensity with electrical stimulation (indicated in yellow). Dashed light green line is the mean APG-2 intensity without electrical stimulation. Shaded areas are standard deviation. (n = 60 cells for each).

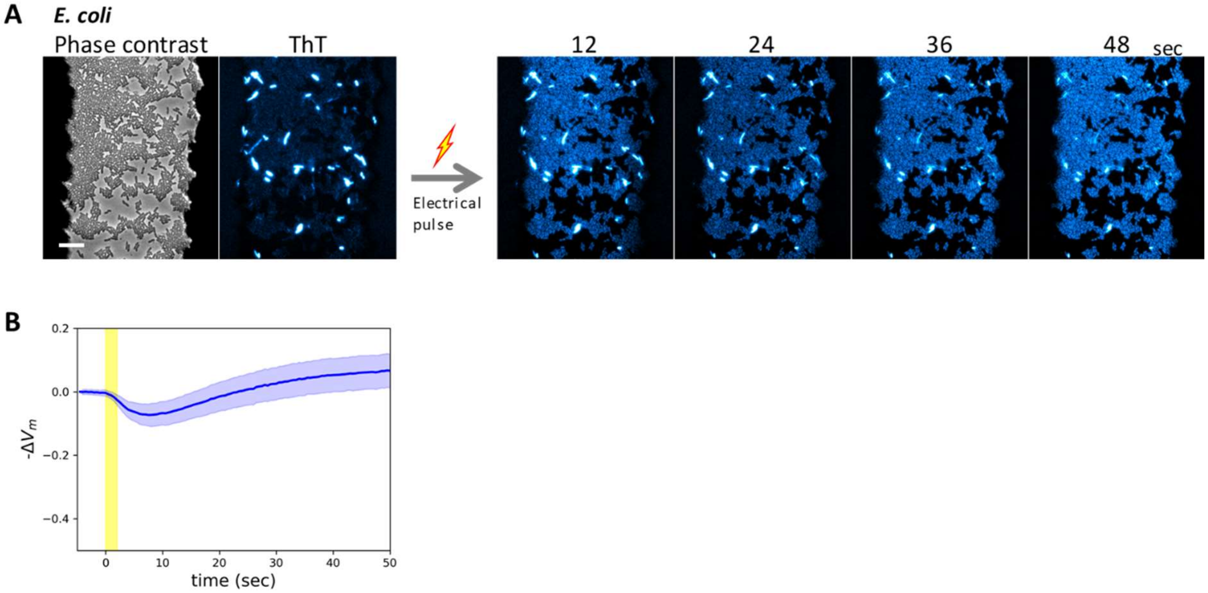

Figure S9 ***E. coli*** **cells also exhibit hyperpolarization response to an electrical stimulation. A)** Film-strip images of ThT fluorescence in *E. coli* before and after an electrical stimulation. Majority of cells hyperpolarize, while a small subpopulation of bright cells at resting state depolarize in response to an electrical stimulation. **B)** Mean membrane potential change (solid line) and standard deviation (shaded region). Yellow depicts the electrical stimulation.

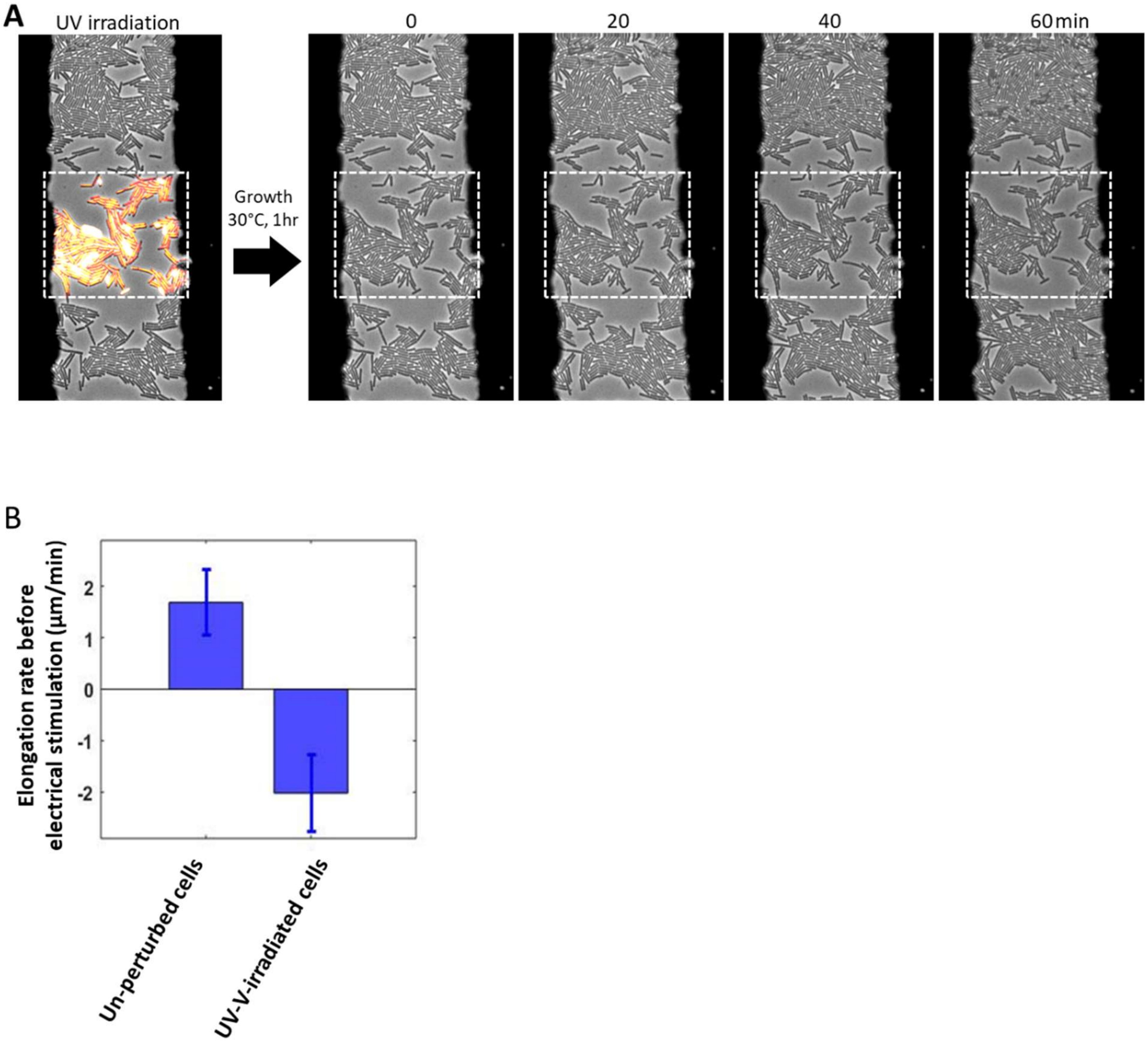

Figure S10 UV-V irradiation inhibit the growth of *B. subtilis* cells. A) Growth suppression by UV-V irradiation was confirmed by phase-contrast time-lapse microscopy. The film-strip images correspond to Fig. 2A. B) Quantification of elongation rates for unperturbed and UV-V irradiated cells (n = 15 cell for each).

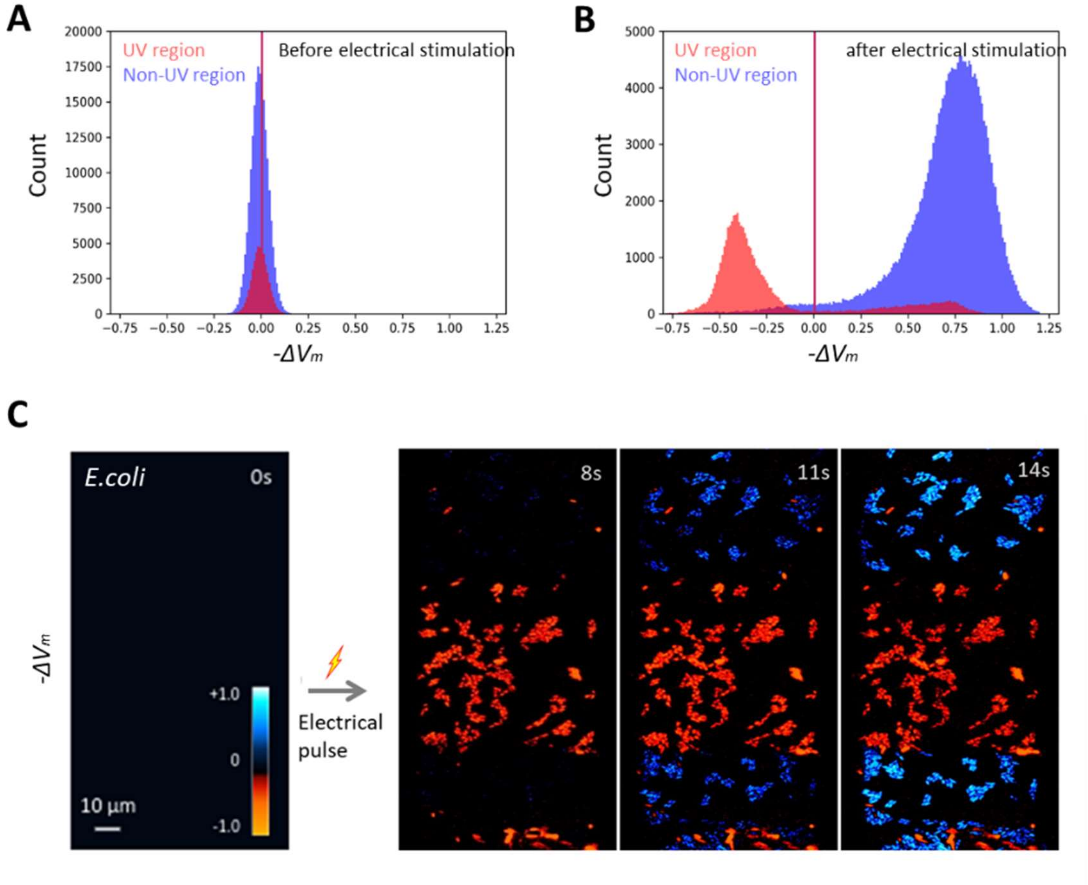

Figure S11 Response differentiation between unperturbed and UV-V irradiated cells.

A, B) Histogram of the membrane potential A) before and B) after electrical stimulation (*B. subtilis*). Blue and red are unperturbed and UV-irradiated regions, respectively. C) Membrane potential response dynamics of *E. coli* cells unperturbed and UV-irradiated cells.

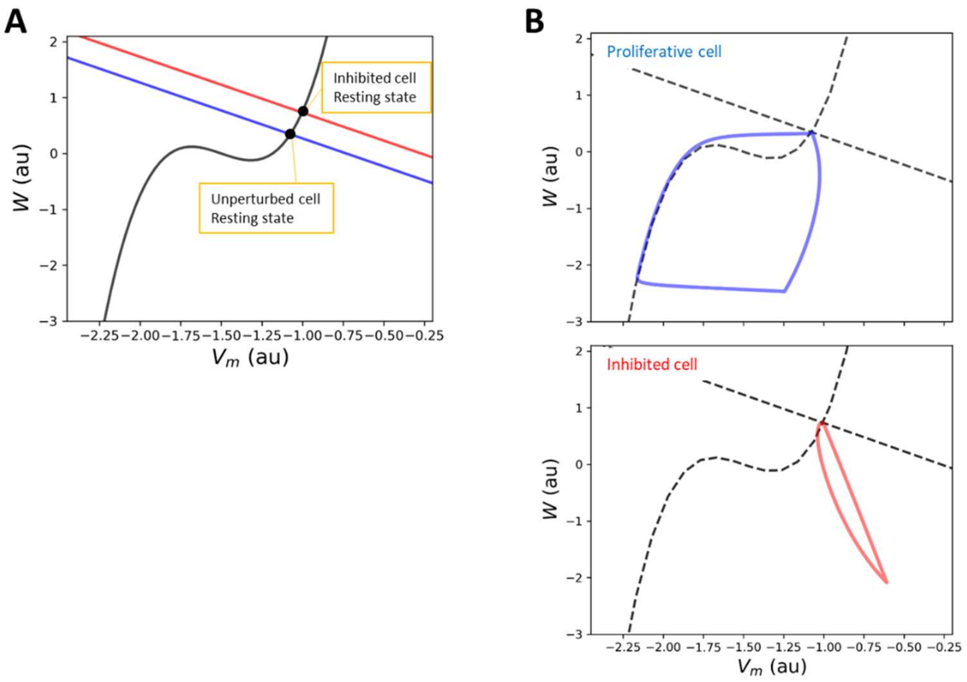

Figure S12 Simulations of the FHN bacteria model in Vm-W phase space. A) Nullcline of Vm is drawn in black. Nullclines of W with the parameters for proliferative and inhibited are drawn in blue and red, respectively. The stable equilibrium points of the system (resting state) are shown by black circles. B) Phase-space trajectories of the simulations for proliferative (blue) and inhibited (red) cells.

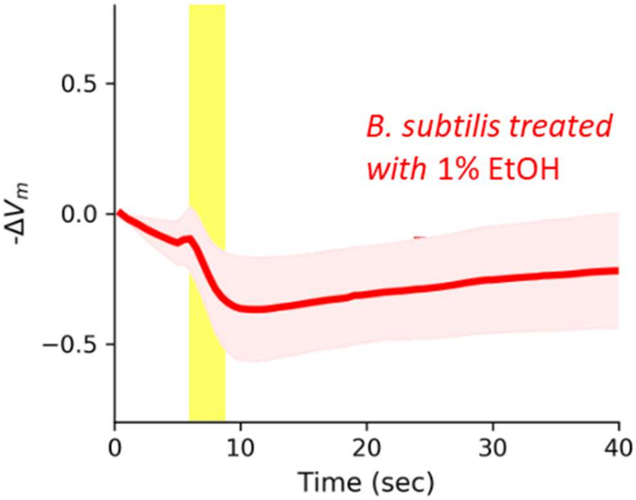

Figure S13 Cells treated with 1% Ethanol depolarize when stimulated by an electrical pulse. Membrane potential dynamics with an application of electrical stimulus (indicated in yellow). Solid line shows the mean intensity and shaded is standard deviation. (n = 60 cells)

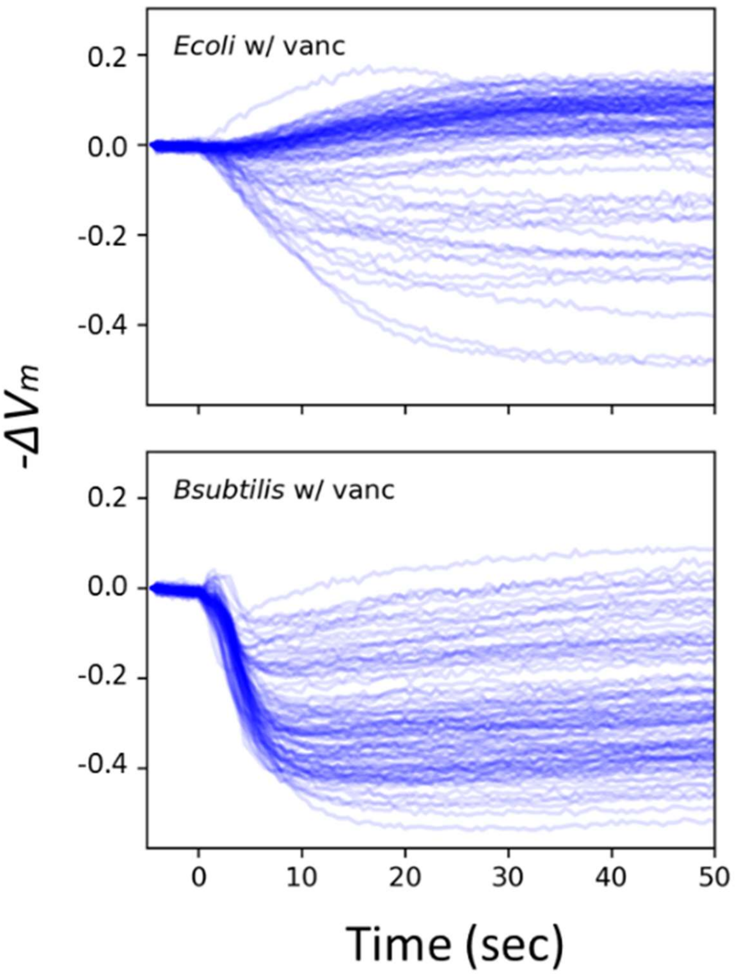

Figure S14 ***E. coli*** **and** ***B. subtilis*** **can be differentiated based on their response dynamics to an electrical stimulus.** Individual cells' response dynamics to an electrical stimulation. The data correspond to Fig. 4E.

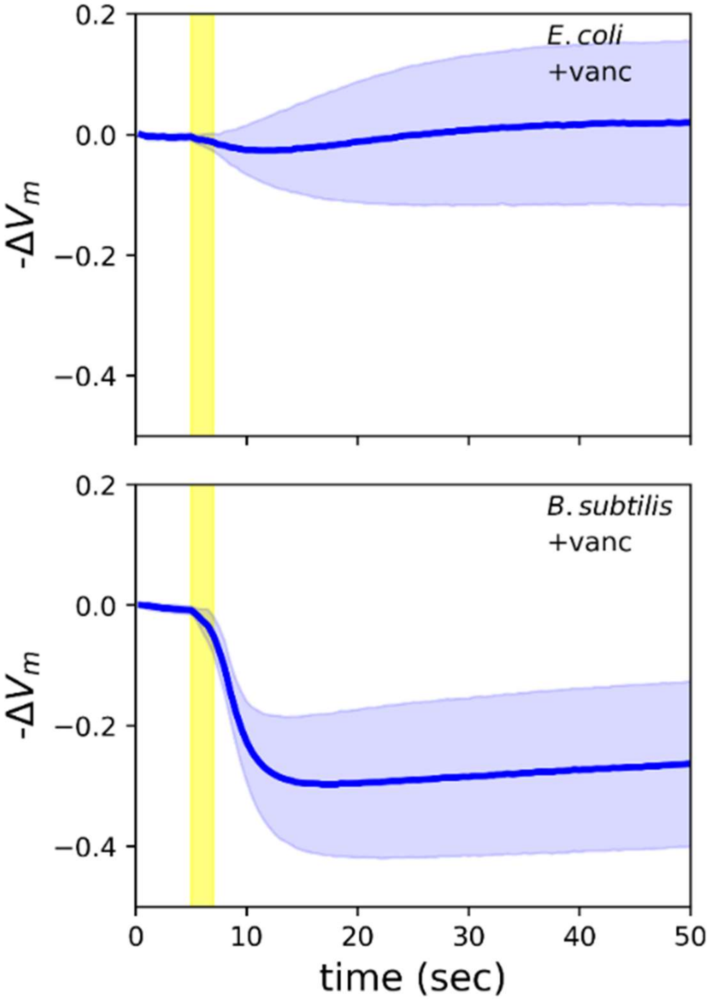

Figure S15 Membrane potential dynamics of *E. coli* and *B. subtilis* cells treated with vancomycin as mono-culture. Solid lines are mean intensity and shaded are standard deviation. (n = 20 cells for each).

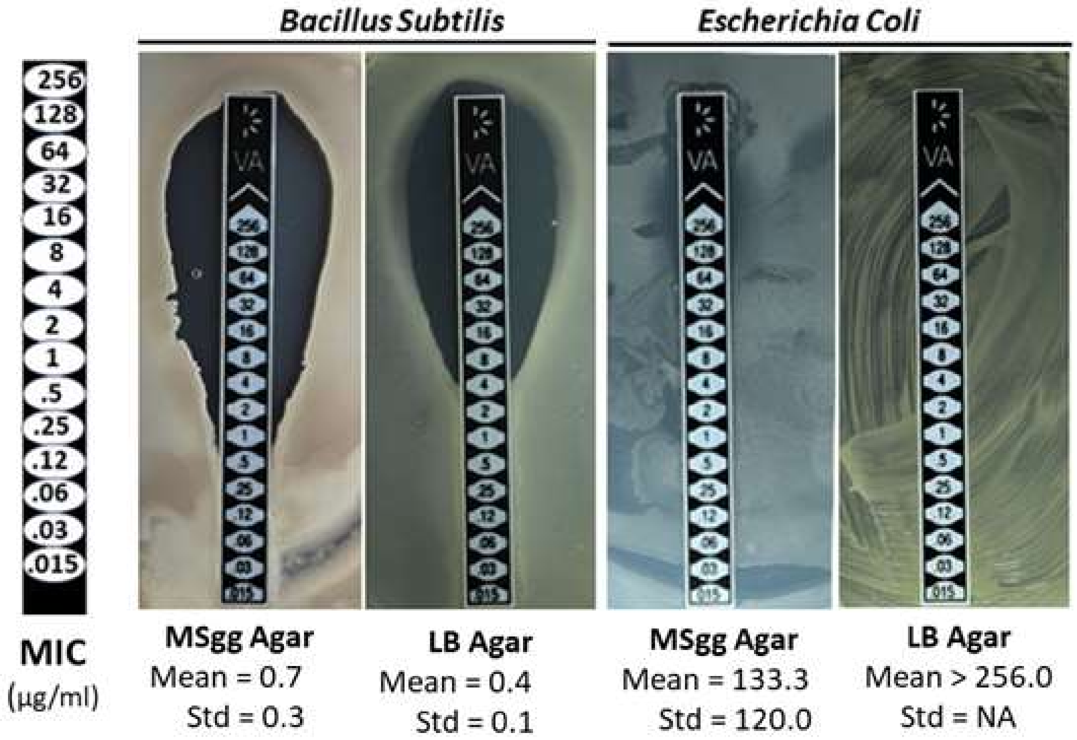

Figure S16 Minimal inhibitory concentration (MIC) for vancomycin against *B. subtilis* and *E. coli* on LB or MSgg agar plates. MIC was determined by reading the edge of clearance zone. The experiments were repeated three times.

Table S1

Strain ID	Specie	Genotype/Plasmid	Source
MA-002	*B. subtilis*	NCIB3610, wildtype	Lab Stock (7)
MA-020	*B. subtilis*	NCIB3610, amyE:: $P_{hyperspank}$-YFP, $spec^R$	Gift from GM Suel (UC San Diego) (8)
MA-627	*B. subtilis*	NCIB3610, yugO:: neo^R	Gift from A Prindle and GM Suel (UC San Diego) (2)
MA-727	*E. coli*	CGSC4401 EMG2 K12, wildtype	Gift from A Pires-daSilva (University of Warwick)
MA-946	*E. coli*	CGSC4401 EMG2 K12, pGEX-6P1-mCherry	This work.

Additional references

1. Ehrenberg B, Montana V, Wei MD, Wuskell JP, Loew LM (1988) Membrane potential can be determined in individual cells from the nernstian distribution of cationic dyes. *Biophys J* 53(5):785–794.
2. Prindle A, et al. (2015) Ion channels enable electrical communication in bacterial communities. *Nature* 527(7576):59–63.
3. Liu J, et al. (2017) Coupling between distant biofilms and emergence of nutrient time-sharing. *Science* 356(6338):638–642.
4. Humphries J, et al. (2017) Species-Independent Attraction to Biofilms through Electrical Signaling. *Cell* 168(1–2):200–209.e12.
5. Kennard AS, et al. (2016) Individuality and universality in the growth-division laws of single E. Coli cells. *Phys Rev E* 93(1). doi:10.1103/PhysRevE.93.012408.
6. Harris LK, Theriot JA (2016) Relative rates of surface and volume synthesis set bacterial cell size. *Cell* 165(6):1479–1492.
7. Asally M, et al. (2012) Localized cell death focuses mechanical forces during 3D patterning in a biofilm. *Proc Natl Acad Sci* 109(46):18891–6.
8. Cağatay T, Turcotte M, Elowitz MB, Garcia-Ojalvo J, Süel GM (2009) Architecture-dependent noise discriminates functionally analogous differentiation circuits. *Cell* 139(3):512–22.
